# MicroRNA-Regulated Gene Delivery Systems for Research and Therapeutic Purposes

**DOI:** 10.3390/molecules23071500

**Published:** 2018-06-21

**Authors:** Bijay Dhungel, Charmaine A. Ramlogan-Steel, Jason C. Steel

**Affiliations:** 1 Gallipoli Medical Research Institute, Greenslopes Private Hospital, 102 Newdegate Street, Brisbane, QLD 4120, Australia; b.dhungel@uq.edu.au; 2 Faculty of Medicine, University of Queensland, 288 Herston Road, Herston, Brisbane, QLD 4006, Australia; c.ramlogansteel@uq.edu.au; 3 University of Queensland Diamantina Institute, Translational Research Institute, 37 Kent Street, Woolloongabba, QLD 4102, Australia; 4 Layton Vision Foundation, Translational Research Institute, 37 Kent Street, Woolloongabba, QLD 4102, Australia; 5 OcuGene, Translational Research Institute, 37 Kent Street, Woolloongabba, QLD 4102, Australia

**Keywords:** gene delivery, gene therapy, targeted transgene expression, microRNA, post-transcriptional targeting

## Abstract

Targeted gene delivery relies on the ability to limit the expression of a transgene within a defined cell/tissue population. MicroRNAs represent a class of highly powerful and effective regulators of gene expression that act by binding to a specific sequence present in the corresponding messenger RNA. Involved in almost every aspect of cellular function, many miRNAs have been discovered with expression patterns specific to developmental stage, lineage, cell-type, or disease stage. Exploiting the binding sites of these miRNAs allows for construction of targeted gene delivery platforms with a diverse range of applications. Here, we summarize studies that have utilized miRNA-regulated systems to achieve targeted gene delivery for both research and therapeutic purposes. Additionally, we identify criteria that are important for the effectiveness of a particular miRNA for such applications and we also discuss factors that have to be taken into consideration when designing miRNA-regulated expression cassettes.

## 1. Introduction

Cell/tissue specific gene delivery is important not only for gene therapy but also to study a range of biological processes within a defined cell population. Targeted gene delivery can be achieved either by application of the gene delivery vector at a specific site (optimizing route of administration), by modification of the vector, or by modification of a transgene by exploiting gene regulatory elements. Even though vector application at a particular site of interest can achieve some levels of targeting [1], the suitability of this method for targeted therapeutic purposes is limited by potential tissue injury and transgene expression in off-target cells [2,3]. Modification of the delivery vehicle, also referred to as transductional targeting, aims to limit the vector entry into target cells by modification of the capsid as in the case of viral vectors [4,5,6,7]. A number of strategies including usage of alternate serotypes [8], insertion of antibodies or bi-specific fusion proteins with targeting ligands, and capsid engineering either by directed evolution or rational design have been successfully used in transductional targeting [5,9]. However, several limitations exist ranging from technical difficulties in manufacturing efficient targeted vectors to problems with manufacturing high quantities of modified vectors when attached to fusion proteins. Additionally, an absolute reversal of natural viral tropism might not be practical leading to some off-target expression of transgene. Another approach that has been used for cell/tissue specific gene delivery is the modification of the therapeutic cassette by transcriptional targeting [10,11]. However, transcriptional targeting with tissue specific promoters is limited by the availability of efficient promoters that can effectively limit transgene expression in the corresponding tissue and/or express and maintain adequate levels of transgene expression [4,12]. Furthermore, promoters in gene therapy vectors often fail to recapitulate the activity of endogenous promoters [13]. Moreover, a combinatorial regime incorporating multiple target layers may provide a stringently controlled targeting platform required for certain applications.

**Figure 1:**
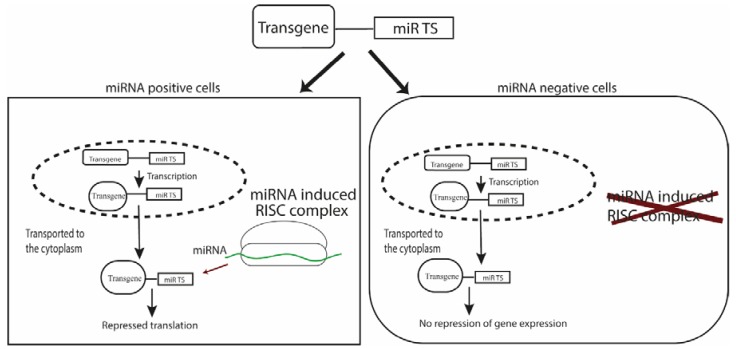
Principle of miRNA mediated regulation of transgene. Construction of miRNA-regulated gene delivery platform for negative targeting is accomplished by incorporation of the binding site (TS) of a miRNA expressed in the target cell/tissue. Endogenous miRNA expressed by the target inhibits transgene expression at post-transcriptional level, whereas transgene expression in non-target cells remains unaffected.

In the past decade, post-transcriptional targeting by exploiting endogenous microRNAs (miRNAs) has emerged as a powerful tool for targeted gene delivery. miRNAs are short, untranslated, regulatory RNA molecules that tightly regulate the expression of a gene by binding to its target sequence (TS) present in the corresponding messenger RNA (mRNA) [14,15]. The inclusion of TSs of endogenous miRNAs, expressed in a particular cell/tissue type, into the UTR of a transgene in a gene vector forms the basis of post-transcriptionally targeted gene delivery (Figure 1). In contrast to positive targeting achieved with tissue specific promoters, miRNA-based targeting is negative as the miRNA TSs are incorporated in expression vectors, thus, cells expressing the corresponding miRNA is detargeted. Efficient miRNA mediated transgene regulation is dependent on the properties of candidate miRNA, binding sites (TSs) as well as the cellular machinery. It is important to consider that not all miRNAs might be useful for detargeting purposes and experimental validation is required for individual applications. This review summarizes studies that have used endogenous miRNA to achieve cell/tissue specific targeting, explores the applications of this method and finally provides workflow for preliminary validation of a candidate miRNA for detargeting a particular cell/tissue type.

## 2. Biogenesis and Mechanism of Action of miRNAs

**Figure 2:**
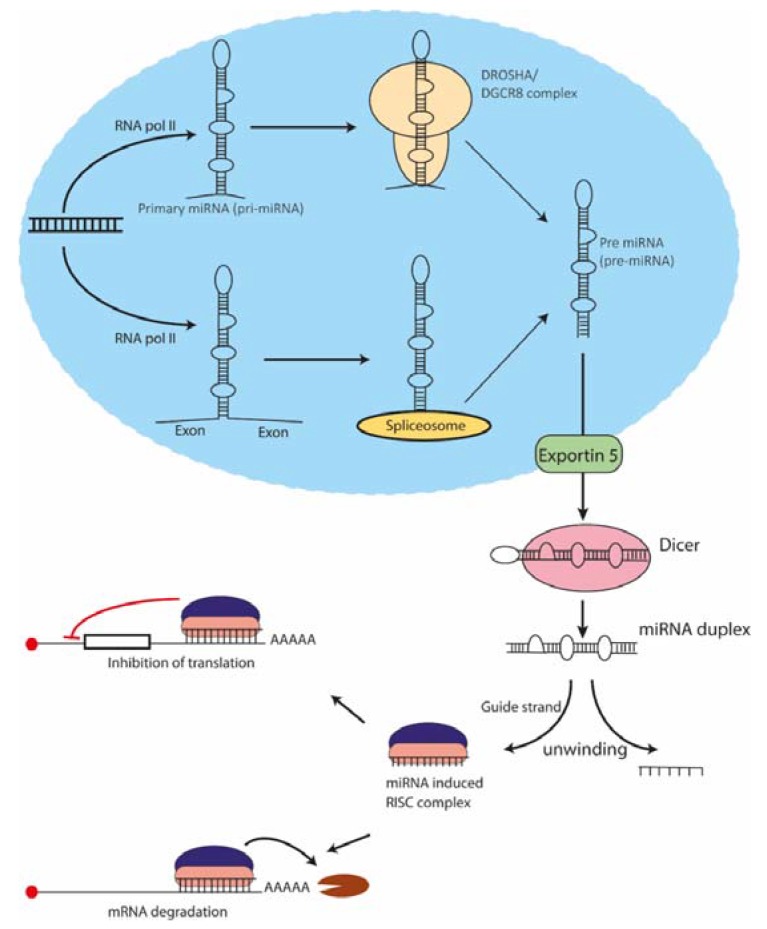
Biogenesis and mechanism of action of miRNAs. miRNAs are produced by two major pathways: canonical and non-canonical pathways. In the former, transcription by RNA pol II results in the formation of primary miRNAs (pri-miRNAs) that are cleaved by RNaseIII enzyme Drosha producing precursor miRNAs (pre-miRNAs) of approximately 70 nucleotide length. Transport molecule exportin 5 then exports pre-miRNAs to the cytoplasm, where they are further processed by another RNaseIII enzyme Dicer and mature miRNA duplex is formed. Following unwinding of the duplex, the guide strand is loaded into a complex along with Argonuate proteins forming an miRNA-induced silencing complex (miRISC). The the miRISC complex to then guided to the messenger RNA transcripts via complementary base pairing between the miRNA and its target sequence (TS) present in the transcript. Finally, depending on the nature of base pairing and other cellular factors, either inhibition of translation or degradation of mRNA occurs.

miRNAs are short, untranslated RNA molecules that regulate the expression of a gene at the post-transcriptional level by binding to a particular sequence that is present in the corresponding mRNA [16]. It is well established that miRNAs are involved in almost every aspect of cellular function, thus playing important roles in development, homeostasis and disease development and/or progression [17]. The canonical miRNA biogenesis involves transcription by RNA polymerase II in a majority of miRNAs followed by Drosha (RNase III enzyme) processing, which produces an approximately 70 nucleotide long precursor miRNA (pre-miRNA) that is transported to the cytoplasm via Exportins [18,19,20,21,22,23]. In the non-canonical pathway of miRNA production, pre-miRNAs are produced via splicing, thus avoiding Drosha action [24]. In the cytoplasm, another RNase III enzyme Dicer cleaves the pre-miRNA to produce mature miRNA, which forms a miRISC (miRNA-associated RNA-induced silencing complex) with the Argonuate protein [19]. RISC complex is then guided to messenger RNA via base pairing with the target sequence (TS) of the miRNA. Perfect complementarity at nucleotides 2–8 in the 5’- end of the miRNA is essential for a successful action of the RISC complex [25,26] (Figure 2). Depending on the extent of complementarity and features of the sequences around the TSs, gene expression is repressed either by inhibition of translation or by cleavage of the corresponding mRNA [27].

## 3. miRNA for Targeted Gene Delivery and Its Applications

### 3.1. Targeted Gene Expression for Research and Therapy

**Figure 3:**
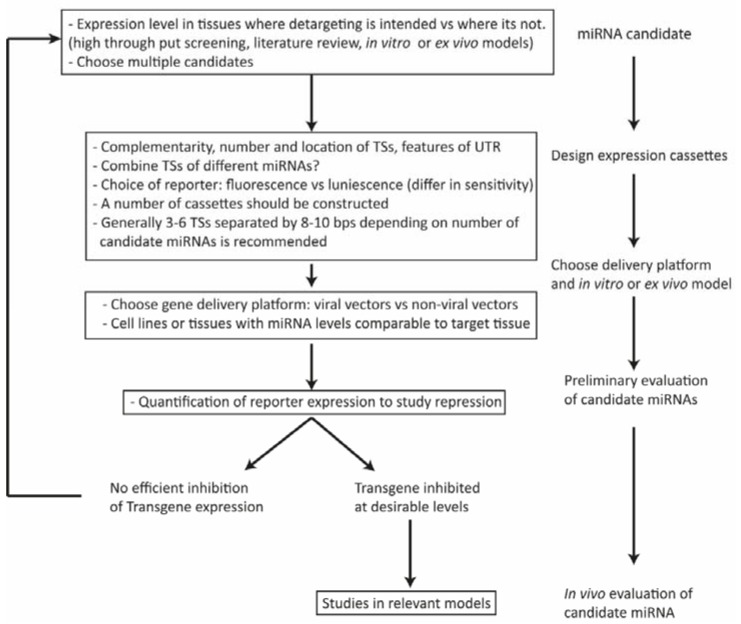
Selection and validation of candidate miRNAs for targeted gene delivery: Depending on the target site (TSs), and nature of application, a few candidate miRNAs are chosen. Generally, miRNAs expressed at high levels in the target cells/tissues whereas at low levels in non-target sites are selected. Generally, the process of optimization of expression cassettes incorporating TSs of candidate miRNAs should include 3–6 TSs separated by 8-10 bps, in case of multiple miRNAs being used in the same cassette, spacing could be decreased. Choosing an appropriate in vitro or ex vivo models expressing candidate miRNAs at a level comparable to target site can bypass the need of in vivo models for preliminary studies. After a successful preliminary evaluation targeted cassettes may be tested in vivo for targeting efficacy, which is followed by the intended application of the targeted delivery system.

The first step in post-transcriptionally targeted gene delivery is choosing an appropriate miRNA that is dictated by the nature of the application (Figure 3). It is important to consider whether a particular study requires disease-specific dysregulated miRNA to prevent off-target effects in normal tissues or detargeting of a certain cell/tissue suffices. For instance, miRNA122a, expressed exclusively in liver and downregulated in hepatocellular carcinoma (HCC), can be utilized for hepatocyte detargeting and thus be incorporated into vectors for targeted gene therapy of HCC [28,29,30,31]. miRNA122a binding sites have been utilized to target other cell/tissue types including cardiac [32], melanoma [33], and adipose tissues [34] to reduce off-target effects in the liver and/or to reduce liver-tropism of the vector. Targeted suicide gene therapy with herpes simplex virus thymidine kinase (HSV-TK) for glioma has been shown to be efficient when incorporating miRNA128 binding sites in the vector [35]. Similar to miRNA122a for HCC, miRNA128 is significantly downregulated in glioma when compared to peripheral tissues, providing a rationale for detargeting normal brain tissue [35]. Targeting of cancer stem cells (CSCs) within a tumor may also be possible with miRNA TSs, as shown by Dhungel et al*.,* where hepatocellular CSCs expressing reduced levels of miRNA122a could be targeted and killed with miRNA122a-regulated cytosine deaminase suicide gene therapy [36]. Recently, miRNA-responsive clustered regularly interspaced short palindromic repeat (CRISPR) and CRISPR-associated systems (Cas) systems have been developed by including TSs of miRNAs at the 3’-UTR of Cas9 mRNA [37]. Incorporating TSs of miRNA21 and 302 at the 3’-UTR of Cas9 mRNA, the investigators obtained attenuated Cas9 activities in HeLa (positive for miRNA21) and induced pluripotent stem cells (miRNA302 positive) respectively. This study provides ground works for precisely controlled cell targeted genome engineering. Some of the tissue/organ enriched candidate miRNAs are presented in Table 1. 

### 3.2. Studying Cell Lineage and Differentiation State

Applications requiring the tracking of a specific population within a mixture of cell types often require miRNAs that are lineage- or differentiation-stage-specific [38]. For instance, let7a, which is specific to pluripotent cells, was used to track the reprogramming of somatic cells to induced pluripotent stem cells (iPSCs) [39]. Furthermore, this particular study reported a system for positive selection of pluripotent stem cells from patients with Rett syndrome and Parkinson’s disease using let7 controlled neomycin resistance gene delivery [39]. Similarly, fluorescence sorting controlled by pluripotent specific miRNA292 was used to separate embryonic stem cells (ESs) from differentiated cells as well as neural stem cells (NSCs). This fluorescence sorting method was subsequently used to isolate neural progenitors from differentiated ESs in order to address the issue of graft rejection and tumor development resulting from contamination of immature cells in predifferentiated cell suspensions after transplant [40]. To selectively target inhibitory neurons in the cortex of the brain, Sayeg et al*.* incorporated TSs of miRNAs 128, 221, and 222 which are expressed at high levels in their excitatory counterparts [41]. By studying the co-localization of markers of excitatory and inhibitory neurons and reporter controlled by the aforementioned miRNAs, they observed both brain tissue as well as neuron-specific targeting with their system [41]. In a similar approach to detarget a specific tissue/compartment within an organ, Brown et al. exploited TSs of miRNAs 122a and 142 to restrict transgene expression in hepatocytes and Kupffer’s cells respectively, while uninhibited transgene expression was observed in other cells within the liver [38].

### 3.3. Redirecting Tropism of Oncolytic Viruses and Construction of Safer Vaccines

Another important application of miRNA mediated regulation of transgene expression is to control tropism of tumor specific oncolytic viruses (OVs) [42]. Viral proteins are highly immunogenic and can lead to inflammation and cell death if expressed in normal cells. Controlled OV replication without attenuation can be achieved by using miRNA TSs [43]. For instance, oncolytic adenovirus with miRNA TS controlled E1A gene displayed superior antitumor activity and prolonged survival in glioma mouse model when compared to attenuated adenovirus ONYX-015 with deleted E1B [44]. Multiple tissue detargeting of the liver, brain, and gastrointestinal tract has been achieved for oncolytic measles virus containing TSs of miRNAs 122a, 7, and 148a respectively [45]. Similarly, endogenous expression of miRNA125 [46] and let7 [47] was used to control the replication of vesicular stomatitis virus (VSV). Conditionally replicating oncolytic adenovirus have also been designed by utilizing TSs of a number of miRNAs including miRNA122a [48,49], miRNA199a [50], miRNA143 [51], miRNA145 [51], let7a [51], miRNA148a [52] and miRNA216a [52]. Similar approaches have been utilized to control the replication of other oncolytic viruses such as herpes simplex virus [29], vaccinia virus [53], and Semliki forest virus [54]. A similar approach of attenuating viruses by incorporating appropriate miRNA TSs can increase the safety of viral vaccines [55]. This approach has been validated in poliovirus with miRNAs 124 and let7a [56], influenza A virus with miRNAs 21 [57], 124 [58], 93 [58], and let7b [59], flavivirus with miRNAs 124 [60], 184 [61], 128 [62], let7 [60,62] and 275 [61], and dengue virus with hematopoietic specific miRNA142 [63].

### 3.4. Repressing Transgene Directed Immune Response

From the point of clinical gene therapy, miRNA-regulated vectors are important in the generation of immune tolerance against the therapeutic gene in order to obtain a stable and long-term gene expression [64]. The clearance of transgene expressing cells by the immune system represents one of the biggest obstacle for long-term gene therapy [65,66]. Even though long-term transgene expression has been achieved in immune privileged organs like the eye [67] and brain [68,69], applications requiring interventions in immune competent organs require methods to induce immune tolerance against the therapeutic gene. Induction of cellular immune response against the transgene and the clearance of transgene expressing transduced cells is primarily the result of naïve T cell priming by professional antigen presenting cells (APCs) including macrophages and dendritic cells. Using miRNA142, which is expressed by cells of the hematopoietic lineage including APCs, reduced reporter expression was observed in APCs and macrophages, resulting in a stable transgene expression [70]. Similarly, lower clearance of factor IX expressing cells transduced cells was observed in a mouse model of hemophilia B when the expression cassette was regulated by miRNA142 TSs [38,71]. More recently, lentivirus expressing factor VIII under the regulation of miRNAs 122a, 142, and 126 (detargeting hepatocytes, hematopoietic cells, and plasmacytoid dendritic cells) was used to maintain long-term expression and obtain therapeutic levels of the gene in a mouse model of hemophilia A [72].

### 3.5. Other Applications

Increasing the yield of viral vectors harboring cytotoxic genes during its manufacture is another area where miRNA-based transgene regulation has been used. Reid et al. showed that HEK293 cells express high levels of miRNA373 and constructed vectors including the TS of this miRNA at the 3’-UTR of cytotoxic genes mitochondrial NADH–ubiquinone oxidoreductase chain and BCL2-associated X protein to prevent expression in HEK293 cells, thereby significantly increasing the overall yield of adeno-associated virus (AAV) [73]. In another study, a non-integrating lentivirus vector expressing conversion factor ABM controlled with TSs of neuron specific miRNA124 was used for direct conversion of fibroblasts into functional neurons providing a safer and clinically relevant cell type conversion system [74]. Gene delivery vectors with TSs of a particular miRNA can also be used as a decoy to saturate or inhibit the miRNA for therapy or to study its biological function [75,76]. Compared to synthetic miRNA inhibitors, which are prone to degradation and require repeated administration to achieve a stable action [77], these vector decoys can provide a more stable option. Moreover, the high production costs and lack of cell/tissue specificity are some limitations of using synthetic miRNA inhibitors that can be addressed using the approached of transcriptionally targeted vector-encoded miRNA inhibitors [75]. In contrast to above mentioned negative targeting, “miRNA-on” systems have also been designed for positive targeting. In this system, two separate cassettes are used, one encoding a repressor protein with miRNA TSs and the other containing transgene and repressor binding site [78]. In the absence of corresponding miRNA, the repressor binding results in inhibition of gene expression whereas mRNA degradation of the repressor in the presence of miRNA releases inhibition of transgene expression. Similarly, induction of endogenous mRNA translation with miRNA TSs has been described for some miRNAs like miR369 and let7 under cellular arrest in G0 phase of cell cycle [79]. Additionally, miRNAs like 138, and 192 bind to TATA box of promoters and promote translation rather than inhibition [80]. It remains to be seen whether similar rules apply to exogenously inserted transgenes.

## 4. Factors Affecting the Design of miRNA-Regulated Gene Delivery Cassettes 

### 4.1. Expression Level of the miRNA

The level at which an endogenous miRNA is expressed by cells/tissues is vital to achieve an efficient inhibition of transgene expression [15]. Real time quantitative PCR methods are usually employed to determine levels of endogenous miRNA and these levels can be compared between target and non-target cells. It has been shown that a threshold of 100 copies of a certain miRNA/pg small RNA is required to achieve repression of a transgene with its TS [38]. However, it should be noted that two different miRNAs expressed at similar levels in the same cell, can have vastly different effects on transgene expression. Similarly, miRNAs that differ by several folds in expression levels, can have similar effects on transgene repression [81]. Additionally, some TSs require higher miRNA levels compared to others, even when perfectly complementary, while others don’t. There are factors, other than expression levels of miRNA, that play important roles in miRNA mediated repression of gene expression including efficient functioning of the RISC complex that is mediated by the action of Ago proteins [82] and the number of competing binding sites of the miRNA in the target cell [83,84]. Nevertheless, for selection of candidate miRNA to detarget transgenes, high endogenous expression levels above a certain threshold is a requirement. An ideal miRNA candidate is expressed at high levels in tissues where detargeting is intended whereas at levels below the threshold where positive expression is required. Comparison of miRNA landscape between the two tissues using high throughput studies can reveal such candidate miRNAs [85,86]. It should be noted that inclusion of TSs of multiple miRNAs in the same vector can allow detargeting of the same or multiple tissues in a cooperative manner [84,87]. Lastly, it has recently been observed that some miRNAs induce translation induction rather than inhibition in quiescence cells, which are cells reversibly arrested at phase G0 of the cell cycle [88]. miRNAs including let7 and miR369 induce upregulation of target mRNAs in the nucleus of G0 cells while repressing its expression in the cytoplasm of dividing ones [79]. Similar observations have been made for miR1 during muscle differentiation, where it enhances mitochondrial DNA translation while suppressing the same in cytoplasm [89]. Although the number of G0 cells in target tissues might not be large enough to have a significant impact in the detargeting outcomes, studying this translational activation of target mRNAs by miRNAs in G0 cells could help better understand the mechanisms of miRNA-induced RNA activation (RNAa) [90].

### 4.2. Configuration, Number, and Position of miRNA Binding Sites

For most of the targeted gene delivery applications, perfectly complementary TSs of miRNA are incorporated at UTR region of a transgene [91]. However, some studies have reported an increased repression activity of endogenous miRNA with imperfectly complementary TSs [84,92]. Geisler et al. observed an increased repression activity of miR206 with 8 out of their 14 mutated TSs suggesting site-directed mutagenesis as a strategy to increase the effectiveness of miRNA mediated transgene regulation [92]. In the same study, the authors observed a cross reaction of miRNA1 having the same seed sequence as miRNA206, which led to inhibition of transgene in unintended tissue. A single nucleotide mutation at the putative AGO2 protein cleavage site inhibited the action of miRNA1 while transgene inhibition by miRNA206 was unhindered [92]. These observations indicate that it is necessary to consider the expression of miRNAs from the same family in the tissues where detargeting is not intended when choosing putative miRNA candidates. In spite of the reports of usefulness of imperfectly complementary TSs, perfect complimentary is preferred for gene therapeutic applications as imperfectly paired TSs inserted exogenously can saturate its corresponding miRNA even at lower concentrations of the transcript, thus affecting normal cellular function of the miRNA [93]. Additionally, perfect complementarity induces RNA degradation allowing miRNA to have a quicker turn over [94,95]. It should be also be noted that some miRNAs including le7i, miR138, miR92, miR181d enhance translation rather than repress it by direct binding with the TATA box motifs present in promoters [80].

Next, the number of binding sites present in the vector plays a vital role in the repression level of a transgene expression. It has been observed that the level of transgene repression increases with the number of TSs available for the miRNA to bind until a certain point, after which none or low effects are observed [91]. For instance, miRNA122a-regulated transgene delivery to HuH7 with one TS was 12.2% lower than with three, while only a 2.6% increase in repression of expression was observed when it was regulated with six TSs [32]. Interestingly, miRNA181-regulated delivery system with two or four TSs showed similar repression levels of transgene in thymocytes but not in peripheral T cells [96]. Although increasing the number of TSs usually increases the level of transgene repression, it should be noted that above a certain threshold of the number of transcripts with the miRNA TSs, target derepression ensues due to saturation, delayed turnover, and/or target RNA-induced miRNA degradation [97,98,99]. To overcome this problem, TSs of different miRNAs expressed in the target tissue at levels above the threshold have been used with success [38,87]. In fact, using multiple TSs of two different miRNAs co-expressed in the target have a better regulatory effect than using TSs of the same miRNA in the context of muscle cells [100]. For targeted delivery platforms, it might be important to compare transgene repression levels achieved by vectors with different numbers of TSs; usually three to six or eight should be considered as a number higher than those can increase the decoy properties of the vector [93,101]. Moreover, having multiple TSs may not be suitable for vectors like AAV with a limited packaging ability [102] as well as increase the possibility of the formation of secondary structures reducing the accessibility of TSs to RISC complexes [103]. 

Similarly, the space between the TSs has been shown to impact the action of miRNAs [104]. We and others have observed an efficient transgene repression even without spacers for perfectly complementary TSs [36,105,106], however, when using imperfectly complementary TSs and achieving best cooperative effects of multiple RISCs, not having some space between the TSs might cause steric hindrances and affect accessibility of target sites reducing transgene inhibition [84,106,107]. Again, long spacers might increase the probability of formation of secondary structures. Generally 3–6 TSs separated by 4–10 nucleotides is recommended, although this number can be lowered when using TSs of multiple miRNAs in the same cassette. The accessibility of miRNA TSs to the RISC complex also depends on the sequences surrounding the binding sites [103]. TSs should be incorporated at sites with a minimal chance of secondary structure formation (is usually the 3’-UTR) [108], even though TSs have been incorporated at 5’-UTR successfully for some miRNAs [48,109]. It has been observed that the efficacy of a binding site increases with the local AU content within the UTRs [103], whereas TSs too close to the stop codon are found to be less effective [100].

## 5. Conclusions

The ability to limit the expression of a therapeutic gene at specific sites is a prerequisite for targeted clinical gene therapy; similarly, regulated reporter expression is needed for studying a range of biological processes within defined cell populations. miRNA mediated post-transcriptional regulation of gene expression is an extremely powerful and versatile tool to achieve targeted gene expression. Unlike the delivery of therapeutic miRNAs, which can cause adverse side effects and possible off-target effects affecting normal cellular physiology, the use of miRNA binding sites (TS) to inhibit the expression of a therapeutic gene in defined cell types harnesses post-transcriptionally targeted gene regulatory machinery in a safe and effective manner [110].

After the pioneering studies performed by Brown et al. [38,70], TSs of several miRNAs have been used to achieve cell/tissue specific inhibition of transgene expression. The applications of this system have ranged from inhibition of transgene directed immune response, targeted gene expression for therapeutic purposes, studying lineage and differentiation states of cell populations, redirecting vector tropism to construct safer viral vectors, and novel applications are being engineered. The range of its application when combined with the ease of using this system with virtually any delivery platforms adds to its versatility. Careful selection of candidate miRNA and construction of gene delivery cassettes following certain criteria can provide an efficient targeting platform. 

miRNAs are master regulators of gene expression, exploiting their robustness and versatility by combining them with other targeting strategies like transcriptional targeting with tissue-specific promoters and transductional targeting with capsid-modified viral vectors or surface-modified non-viral vectors can provide a tightly controlled gene expression system which may limit the off target effects of traditional gene therapy, thereby increasing the safety of gene therapy in clinical applications.

## Figures and Tables

**Table 1:** miRNAs specific to or enriched in organs/tissues.

Organ/Tissue Type	miRNA	References
Eye	miR124, miR204, miR181	[1,2]
Heart	miR1, miR206, miR126, miR134, miR133, miR208, miR302	[3,4,5,6,7,8,9]
Brain/Nervous system	miR338, miR219, miR124, miR9, miR218, miR7, miR128, miR125, miR138, miR132, miR212, miR137, miR31, miR127, miR143, miR346, miR708	[10,11,12,13,14,15,16,17,18]
Kidney	miR10, miR192, miR204, miR194, miR215, miR216	[9,19,20]
Liver	miR122a, miR192, miR92a, miR483	[9,20]
Lung	miR126	[9]
Hematopoietic and pluripotent cells	miR126, miR130, miR302, miR292	[21,22,23]
Pancreas	miR216, miR217	[9]
Muscle	miR133, miR1, miR206, miR134, miR193a, miR128a	[9,10]
Immune system	miR150, miR181a, miR155, miR142	[24,27]
